# Mosapride stimulates human 5-HT_4_-serotonin receptors in the heart

**DOI:** 10.1007/s00210-024-03047-1

**Published:** 2024-03-18

**Authors:** Joachim Neumann, Christin Hesse, Britt Hofmann, Ulrich Gergs

**Affiliations:** 1 Institute for Pharmacology and Toxicology, Medical Faculty, Martin Luther University Halle-Wittenberg, Magdeburger Straße 4, 06112 Halle (Saale), Germany; 2 Department of Cardiac Surgery, Mid-German Heart Center, University Hospital Halle, Ernst-Grube-Straße 40, 06097 Halle (Saale), Germany

**Keywords:** Mosapride, Human atrium, Mouse atrium

## Abstract

Mosapride (4-amino-5-chloro-2-ethoxy-N-[[4-[(4-fluorophenyl) methyl]-2-morpholinyl]-methyl] benzamide) is a potent agonist at gastrointestinal 5-HT_4_ receptors. Mosapride is an approved drug to treat several gastric diseases. We tested the hypothesis that mosapride also stimulates 5-HT_4_ receptors in the heart. Mosapride increased the force of contraction and beating rate in isolated atrial preparations from mice with cardiac overexpression of human 5-HT_4_-serotonin receptors (5-HT_4_-TG). However, it is inactive in wild-type mouse hearts (WT). Mosapride was less effective and potent than serotonin in raising the force of contraction or the beating rate in 5-HT_4_-TG. Only in the presence of cilostamide (1 μM), a phosphodiesterase III inhibitor, mosapride, and its primary metabolite time dependently raised the force of contraction under isometric conditions in isolated paced human right atrial preparations (HAP, obtained during open heart surgery). In HAP, mosapride (10 μM) reduced serotonin-induced increases in the force of contraction. Mosapride (10 µM) shifted the concentration–response curves to serotonin in HAP to the right. These data suggest that mosapride is a partial agonist at 5-HT_4_-serotonin receptors in HAP.

## Introduction

**Fig. 1:**
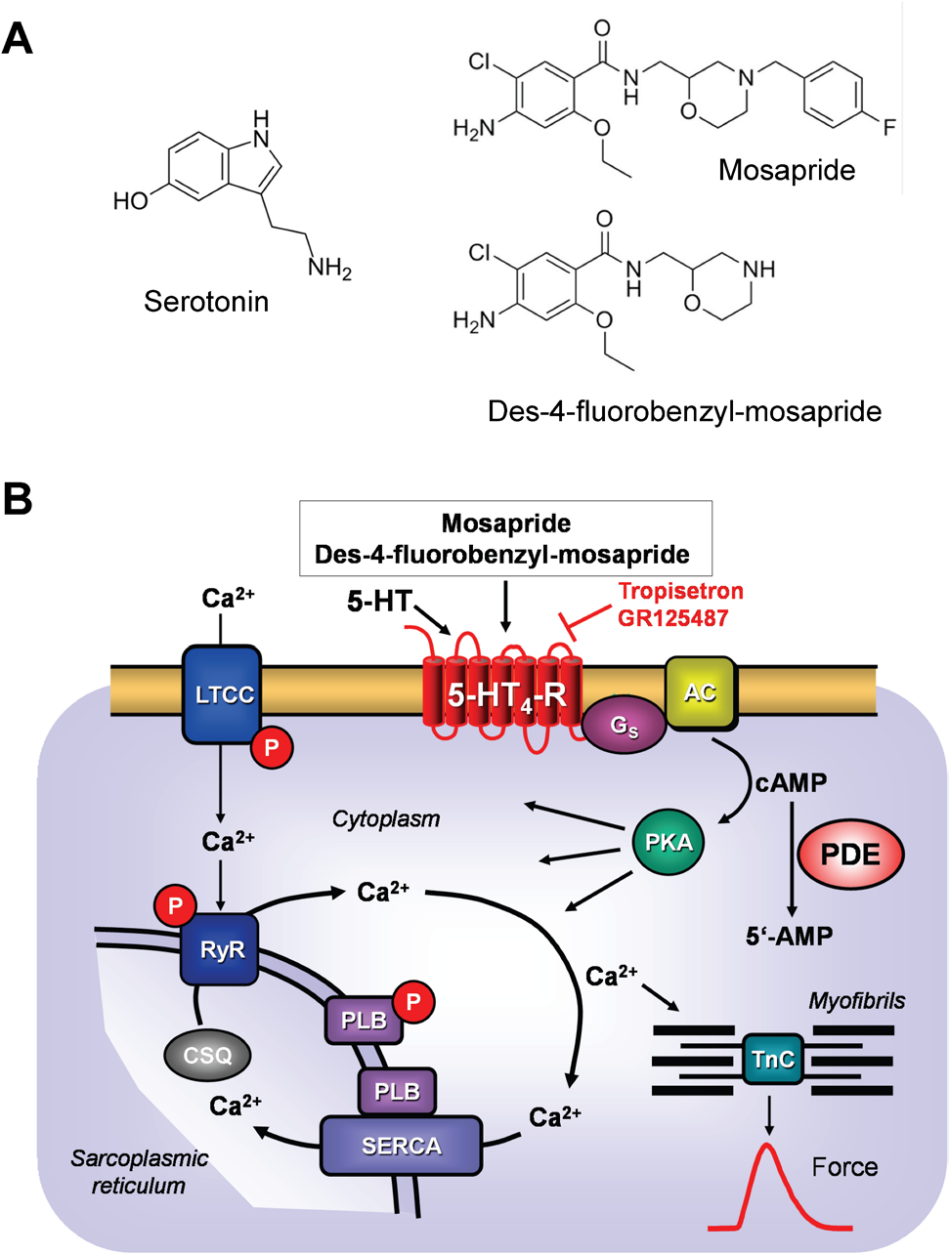
**A** Structural formulae of serotonin, des-fluorobenzyl-mosapride and mosapride. Note the benzamide structure in mosapride, the different side chain of mosapride compared to serotonin and the metabolism of mosapride to its active metabolite des-fluorobenzyl-mosapride. **B** (Scheme): Mechanism(s) of action of serotonin and mosapride in cardiomyocytes. A heptahelical 5-HT_4_-serotonin receptor is depicted in sarcolemma. The agonist serotonin (5-HT) activates the 5-HT_4_-serotonin receptor. Thereby, the stimulatory G-protein (G_s_) augments the ability of adenylyl cyclases (AC) to generate cAMP. This cAMP can activate cAMP-dependent protein kinases (PKA). Thereafter, PKA phosphorylates and activates target proteins like the L-type calcium channel (LTCC) in the sarcolemma and the ryanodine receptor (RyR) in the sarcoplasmic reticulum (SR). Phosphorylation of phospholamban increases the activity of SR-Ca ATPAse (SERCA). Phosphodiesterase (PDE) III converts cAMP to inactive 5′-AMP in the human heart and is inhibited by cilostamide. Mosapride and des-fluorobenzyl-mosapride may activate human cardiac 5-HT_4_-serotonin receptors

Mosapride (Fig. 1A) was developed by a Japanese company (reviewed in: Katoh et al. 2003) and is approved in patients and mainly sold in Japan and some other Asian countries. Mosapride is intended to treat various gastrointestinal diseases (Curran and Robinson 2008). Mosapride has at least one active metabolite (Katoh et al. 2003), called des-fluorobenzyl-mosapride (Fig. 1A), which we studied for comparison. Mosapride acts functionally as a partial agonist compared to 5-HT at 5-HT_4_ receptors in ligand-binding studies (Tsubouchi et al. 2018). Like many other benzamides (e.g. zacopride), mosapride is also an antagonist at 5-HT_3_ receptors (Park and Sung 2019). One had developed mosapride because previous work showed that agonists at 5-HT4 receptors were promising agents for treating gastric diseases (Katoh et al. 2003). However, the authors argued that other drugs that increase gastric motility show agonistic or antagonistic effects at other receptors, in addition to their stimulatory effect on 5-HT_4_ receptors (Katoh et al. 2003). For example, they gave metoclopramide or cisapride (Katoh et al. 2003). For instance, metoclopramide is not only an agonist at 5-HT_4_-receptors but also acts as an antagonist at D_2_-dopamine receptors.

Thus, metoclopramide can lead to Parkinson-like side effects and elevated prolactin levels (Katoh et al. 2003, Athavale et al. 2020). In parallel, soon after cisapride entered the market, cardiac arrhythmias were reported that were explained by the inhibitory action of cisapride on human ventricular potassium channels (Tack et al. 2012). This manifested in prolonging QT interval on surface ECG (e.g. dog: Matsunaga et al. 2011). At least in isolated perfused rabbit hearts, mosapride concentration dependently increased the duration of the QT interval and increased the incidence of early after-depolarisation (Kii and Ito 2002). Prolongation of QT intervals sometimes leads to torsade de pointes and deadly ventricular fibrillation. Mosapride has the advantage of being about three orders of magnitude less potent than cisapride to inhibit ventricular potassium channels and, thus, less likely to prolong the QT interval and induce arrhythmias by this electrophysiological mechanism (rabbit: Carlsson et al. 1997; rat: Kii et al. 2001, dog: Matsunaga et al. 2011). However, another potassium channel that is expressed in the heart, Kv4.3, is also inhibited by mosapride (Sung and Hahn 2013). This may result in a propensity for arrhythmias in vulnerable patients. Indeed, a clinical registry study noted an increased incidence of arrhythmias in patients taking mosapride compared to non-users (Song et al. 2020).

Moreover, the cardiac effects of mosapride might be beneficial. For instance, doxorubicin is an important anti-cancer drug. Its use is limited by the cardiotoxic effects of doxorubicin. For example, doxorubicin leads to arrhythmia and heart failure in humans (Nishiuchi et al. 2022). Interestingly, in mice treated with doxorubicin as a model of human heart failure, an improvement in cardiac contractility was observed when mosapride was given together with doxorubicin (Nishiuchi et al. 2022). This raises the option of starting a clinical trial with mosapride in cancer patients who need chemotherapy that includes doxorubicin. In this regard, it is crucial to study mosapride in the human heart before such trials can be initiated in earnest.

The data in rabbits, rats and dogs are consistent with an inhibitory action of mosapride on potassium channels but tell nothing about the involvement of 5-HT_4_ receptors (Fig. 1B) because 5-HT_4_ receptors are not functionally present in rabbit cardiomyocytes (review: Neumann et al. 2017, 2023a). However, stimulation of 5-HT_4_ receptors in the heart can lead to cardiac arrhythmias (Neumann et al. 2017, 2023a, Keller et al. 2018). Mosapride has never been studied for its functional effects on human 5-HT_4_ receptors in the heart. This fact motivated us to initiate the present study.

All inotropic and chronotropic effects of serotonin are mediated via 5-HT_4_ receptors on human cardiomyocytes (reviews: Kaumann and Levy 2006, Neumann et al. 2017, Neumann et al. 2023a, Fig. 1B). These 5-HT_4_ receptors are lacking in a functional manner in mouse hearts: serotonin does not increase the force of contraction in isolated mouse cardiac preparations from wild-type mice (WT, Gergs et al. 2010, 2013). To facilitate the study of human 5-HT_4_ receptors, we previously established a transgenic mouse with overexpression of this receptor (5-HT_4_-TG) only in the heart, which responds with positive inotropic and positive chronotropic effects to serotonin and other 5-HT_4_ receptor agonists (Gergs et al. 2010; review: Neumann et al. 2017, 2023a, 2023b). Hence, we decided to test whether mosapride would exert positive inotropic and chronotropic effects in this 5-HT_4_-TG and not in littermate WT. If that were the case, one would also expect mosapride to stimulate the 5-HT_4_ receptors in the human heart and thereby increase the force of contraction (Fig. 1B). We used this reasoning with some success in the past. For instance, we found that metoclopramide, cisapride, bufotenin and prucalopride stimulated 5-HT_4_ receptors in the atrium of 5-HT_4_-TG as well as the human atrial preparations (HAP) in vitro (e.g. Keller et al. 2018, Neumann et al. 2021a, 2023b).

Hence, we tested the following hypotheses:

1. *Mosapride increases the force of contraction and spontaneous beating rate in atrial preparations from 5-HT*_*4*_*-TG (and not WT).*
2. *Mosapride and des-fluorobenzyl-mosapride increase the force of contraction in HAP *via* 5-HT*_*4*_* receptors.*

A progress report has been published in abstract form (Neumann et al. 2023c).

## Materials and methods

### Contractile studies in mice

Mice in this study included transgenic mice (CD1 background), where the full-length human 5-HT4 receptor is overexpressed in the heart driven by α-myosin heavy-chain promoter (5-HT_4_-TG). The generation and initial characterisation of these mice at biochemical and functional levels was reported some years ago (Gergs et al. 2010). The initial founder was then crossed in mice of the strain CD-1. For comparison, we used littermate wild-type animals (WT). We used mice of random sex about 130 days of age.

In brief, the right or left atrial preparations in the mice were isolated and mounted in organ baths, as previously described (Gergs et al. 2013; Neumann et al. 1998). The bathing solution of the organ baths contained 119.8 mM NaCI, 5.4 mM KCI, 1.8 mM CaCl_2_, 1.05 mM MgCl_2_, 0.42 mM NaH_2_PO_4_, 22.6 mM NaHCO_3_, 0.05 mM Na_2_EDTA, 0.28 mM ascorbic acid and 5.05 mM glucose. The solution was continuously gassed with 95% O_2_ and 5% CO_2_ and maintained at 37 °C and pH 7.4 (Neumann et al. 1998). The force of contraction was quantified in electrically paced isolated left atrial preparations. The duration of electrical stimulation with a rectangular impulse of direct current was 5 ms. The voltage was 10% higher than necessary to initiate contraction and the stimulation rate was one beat per second (1 Hz). Muscles were stretched such that the maximum basal force was generated and then allowed to stabilise for 30 min before drug application. Spontaneously beating right atrial preparations in mice were used to study any chronotropic effects. The drug application was as follows: After equilibration was reached, mosapride was added cumulatively to the left or right atrial preparations to establish concentration–response curves.

Next, where indicated, serotonin was cumulatively applied to the preparations to compare the efficacy of mosapride and serotonin. We studied WT (*n* = 5) and 5-HT_4_-TG (*n* = 6) from both genders. The average age was 144 days.

### Contractile studies on human preparations

Contractile studies on human preparations were done using the same setup and buffer used in the mouse studies. In brief, the force of contraction was quantified in electrically paced isolated left atrial preparations. The duration of electrical stimulation with a rectangular impulse of direct current was 5 ms. The voltage was 10% higher than necessary to initiate contraction. Muscles were stretched such that the maximum basal force was generated and then allowed to stabilise for 30 min before drug application started. Basal developed force can be seen in the relevant diagrams in this paper labelled with millinewton (mN) in the ordinates under the condition labelled control conditions (Ctr). The samples were obtained from ten male and four female patients aged 45–83. The patients suffered from coronary diseases (two- and three-vessel diseases), atrial fibrillation and hypertension. Drug therapy included metoprolol, furosemide, apixaban, statins and acetylsalicylic acid. The methods used for atrial contraction studies in human samples have been previously published and were not altered in this study (Gergs et al. 2009, 2017). Informed written consent was obtained from all patients.

The drug application was as follows: After equilibration was reached, mosapride was cumulatively added to HAP to establish concentration–response curves. In separate experiments, the first 1 μM cilostamide was given. We waited until a positive inotropic effect on cilostamide had developed and reached a plateau. Then, we constructed a concentration–response curve for mosapride. In some preparations, an antagonist was added (either tropisetron or GR 125487). Then, where indicated, serotonin was cumulatively applied to the preparations. In other experiments, 10 µM mosapride alone was added, and then serotonin was cumulatively applied. These results were compared with separate preparations in which only a concentration response to serotonin was constructed.

### Data analysis

Data shown are the means ± standard error of the mean. Statistical significance was estimated using analysis of variance (ANOVA) followed by Bonferroni’s *t*-test. A *p*-value < 0.05 was considered significant.

### Drugs and materials

( −)-Isoprenaline-( +)-bitartrate, des-4-fluorobenzyl-mosapride (4-amino-5-chloro-2-ethoxy-N-(2-morpholinylmethyl)-benzamide, mosapride (4-amino-5-chloro-2-ethoxy-N-[[4-[(4-fluorophenyl)methyl]-2-morpholinyl]methyl]-benzamide) citrate, serotonin hydrochloride, GR 125487 [1-[2-(methanesulfonamido)ethyl]piperidin-4-yl]methyl 5-fluoro-2-methoxy-1H-indole-3-carboxylate) sulfamate, tropisetron (ICS 205–930, [(1R,5S)-8-methyl-8-azabicyclo[3.2.1]octan-3-yl] 1H-indole-3-carboxylate) hydrochloride and cilostamide (*N*-cyclohexyl-*N*-methyl-4-(1,2-dihydro-2-oxo-6-quinolyloxy) butyramide) were purchased from Cayman (via Biomol, Hamburg, Germany), Selleckchem (Cologne, Germany), Tocris (Wiesbaden-Nordenstadt, Germany) or Sigma-Aldrich (now: Merck, Derieich, Germany), respectively. All other chemicals were of the highest purity grade commercially available. Deionised water was used throughout the experiments. Stock solutions were prepared fresh daily.

## Results

**Fig. 2:**
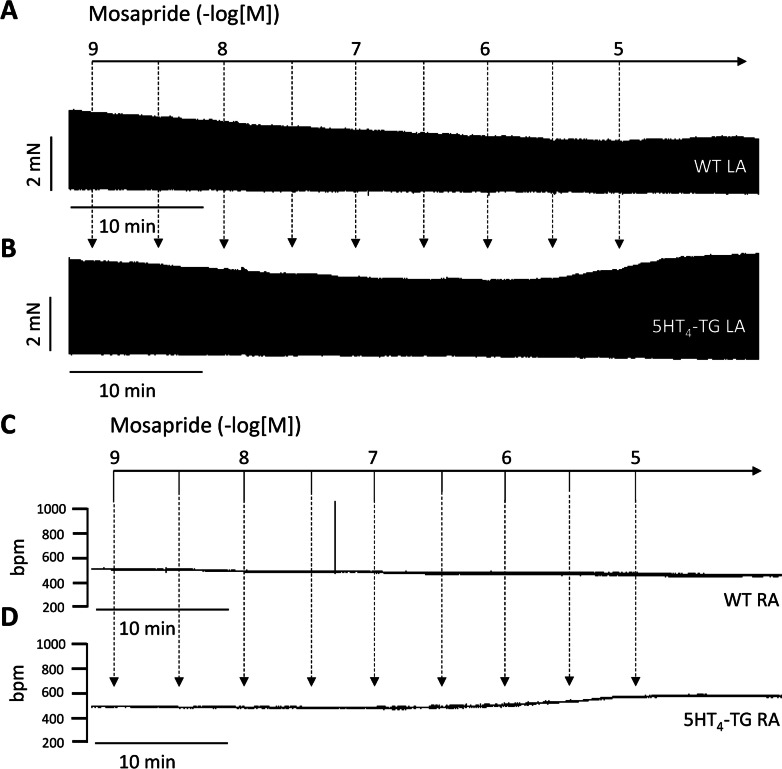
Original recordings in electrically driven left atrial preparations from 5-HT_4_-TG (**B**) or from WT (**A**). Mosapride induced a time- and concentration-dependent positive inotropic effect in 5-HT_4_-TG (**B**) but not WT (**A**). Original recordings of the effect of mosapride in spontaneously beating right atrial preparations from 5-HT_4_-TG (**D**) or from WT (**C**). Mosapride induced a time- and concentration-dependent positive chronotropic effect in 5-HT_4_-TG (**D**) but not WT (**C**). Ordinates in panels **A** and **B** in millinewton (mN). Ordinates in panels **C** and **D** in beats per minute (bpm). Horizontal bars indicate time in minutes (min). Arrows indicate at what time points mosapride was cumulatively applied to the organ bath. Concentrations of mosapride are given in negative logarithmic units in the horizontal arrow in the top

**Fig. 3:**
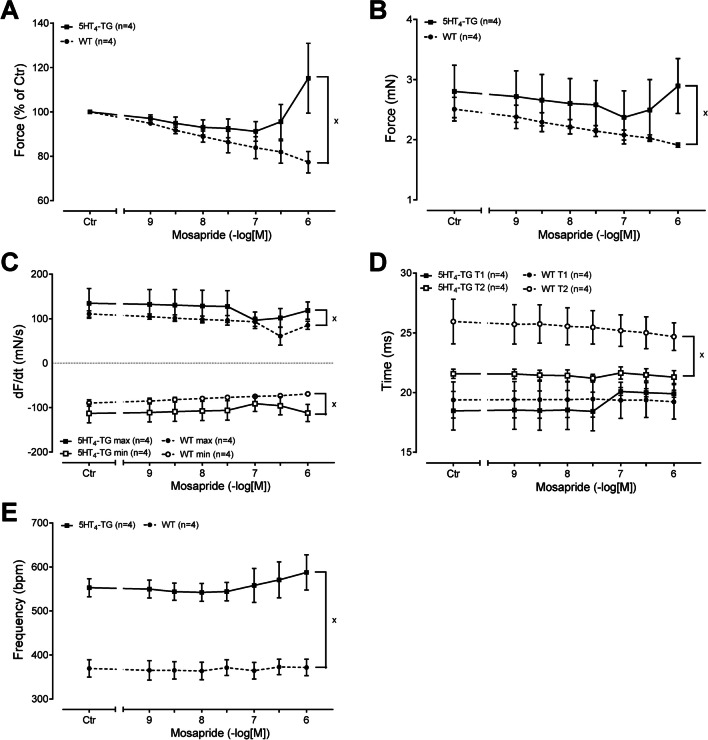
Summarised concentration–response curves for the effect of mosapride on force of contraction in % of pre-drug value (**A**) in mN (**B**). Furthermore, the rate of tension development and the rate of relaxation are given in panel **C**. The effect of mosapride on time to peak tension (T1, **D**) and time of relaxation (T2, **D**). The effect of mosapride on beating rate is given in panel **E**. Ordinates in panels **A** and **B** are in milliseconds (ms). Rate of contraction and rate of relaxation are in panel **C** in mN/s. Contraction times are in ms in panel **D**. Abscissae in panels **A**–**E** indicate concentrations of mosapride in negative logarithm of molar concentrations. Significant differences versus 5-HT_4_-TG are indicated by *x*. Numbers in brackets mean number of experiments

As seen in this original recording, mosapride exerted a concentration- and time-dependent positive inotropic effect in the left atrial preparations from 5-HT_4_-TG (Fig. 2B). In contrast, mosapride failed to raise the force of contraction in the left atrial preparations from WT (Fig. 2A). The latter finding is consistent with our previous research. 5-HT cannot raise force in the atrium from WT (Gergs et al. 2010). The expression of the 5-HT_4_ receptor or the coupling of the 5-HT_4_ receptor is too small to affect contractility in the mouse heart (discussed in Gergs et al. 2010). If mosapride behaves like 5-HT, mosapride should affect the beating rate in right atrial preparations of 5-HT_4_-TG. Furthermore, we noticed a small time- and concentration-dependent positive chronotropic effect of mosapride in right atrial preparations from 5-HT_4_-TG (Fig. 2D), which is plotted in Fig. 3D in an original recording.

In contrast, we detected no positive inotropic effect in the right atrial preparations from WT (Figs. 2C and 3C). We also assessed muscle tension parameters in the left atrium. Several such experiments are summarised regarding the force of contraction measured as the percent of pre-drug value or mN in the left atrium, as seen in Fig. 3A and B. This effect gained statistical significance at 1 µM mosapride. Furthermore, the rate of tension development and the rate of relaxation were not changed in absolute values by mosapride (Fig. 3C). Moreover, we were interested in the effect of mosapride on the times of contraction. It turned out that mosapride did not reduce the time to peak to tension (T1 in Fig. 3D) or the time of relaxation (T2 in Fig. 3D). Finally, mosapride tended to increase the beating rate (Fig. 3E).

**Fig. 4:**
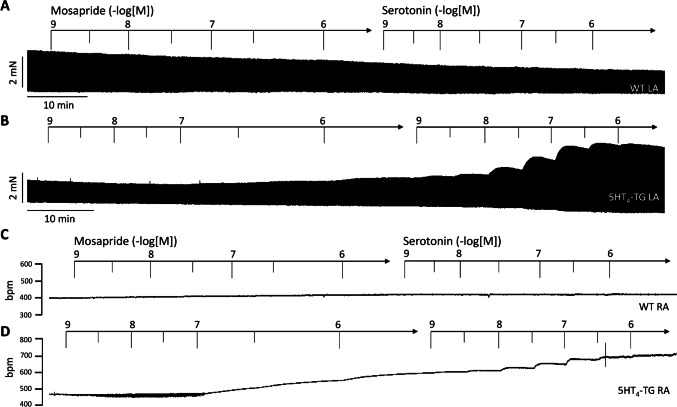
Original recording in mouse left atrial preparation from 5-HT_4_-TG. Mosapride induced a time- and concentration-dependent positive inotropic effect in 5-HT_4_-TG that was followed by a further positive inotropic effect in the additional presence of serotonin (**B**). Original recordings of the effect of mosapride and serotonin in left atrial preparation from WT (**A**). Mosapride induced a time- and concentration-dependent positive chronotropic effect in right atrial preparations from 5-HT_4_-TG that was augmented by additionally applied serotonin in right atrial preparations from 5-HT_4_-T (**D**) but not from WT (**C**). Horizontal bars indicate time in minutes (min). Arrows indicate at what time points mosapride or serotonin were cumulatively applied to the organ bath. Concentrations of mosapride and serotonin are given in negative logarithmic units in the horizontal arrow in the top

**Fig. 5:**
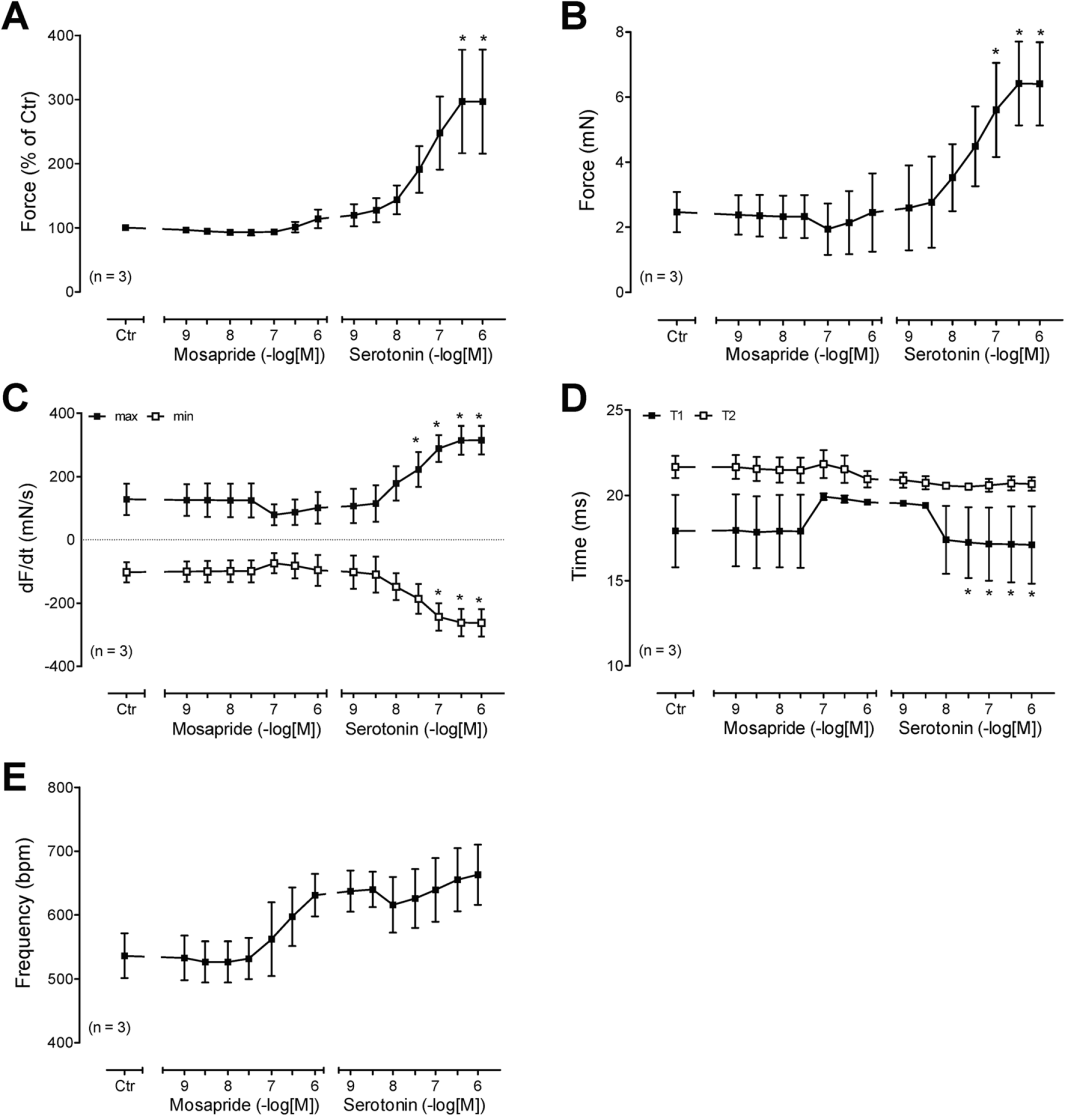
**A** Summarised data for the concentration dependent effect of cumulatively applied mosapride followed by serotonin in left atrial preparations from 5-HT_4_-TG for force of contraction in % of pre-drug value (**A**) in mN (**B**), rate of tension development (**C**: dF/dt_max_), rate of tension relaxation (**C**: dF/dt_min_) both in mN/s and on time to peak tension (T1, **D**) and on time of relaxation (**D**: T2). Ordinates give force of contraction in panel **A** in % of pre-drug value or in panel **B** in mN, the rate of contraction and rate of relaxation in panel **C** in mN/s, the contraction times in panel **D** in milliseconds (ms) and in panel **E** in beats per minute (bpm). Abscissae in panels **A**–**E** indicate concentrations of mosapride and of serotonin in negative logarithm of molar concentrations. Significant differences versus 5-HT_4_-TG are indicated in *x*. Numbers in brackets mean number of experiments

In the original recordings, we depicted that in separate experiments, first applying mosapride and then subsequently applying 5-HT increased the force of contraction further in left atrial preparations from 5-HT_4_-TG (Figs. 4B, 5A and 5B). In contrast, the subsequent application of 5-HT did not increase the force of contraction in WT (Fig. 4A, Gergs et al. 2010, 2013).

Likewise, following mosapride, additionally applied 5-HT increased the beating rate further in 5-HT_4_-TG, suggesting that mosapride is not a full agonist in relation to the beating rate (Figs. 4D and 5E). In contrast, neither mosapride nor serotonin augmented the beating rate in the right atrial preparations from WT (Fig. 4C, Gergs et al. 2010, 2013).

We also assessed muscle tension parameters in the left atrium under these conditions (i.e. Fig. 4B). Several such experiments are summarised regarding the force of contraction measured as the percent of pre-drug value or mN in the left atrium (Fig. 5A, B). This effect gained statistical significance at 0.3 µM serotonin. Furthermore, the rate of tension development and the rate of relaxation were not augmented in absolute values by mosapride, but by subsequent serotonin (Fig. 5C). Moreover, we were interested in the effect of mosapride on the times of contraction. The result was that mosapride did not reduce the time to peak tension (Fig. 5D) or the time of relaxation (T2, Fig. 3D). Finally, serotonin after mosapride further increased the beating rate (Fig. 5E).

**Fig. 6:**
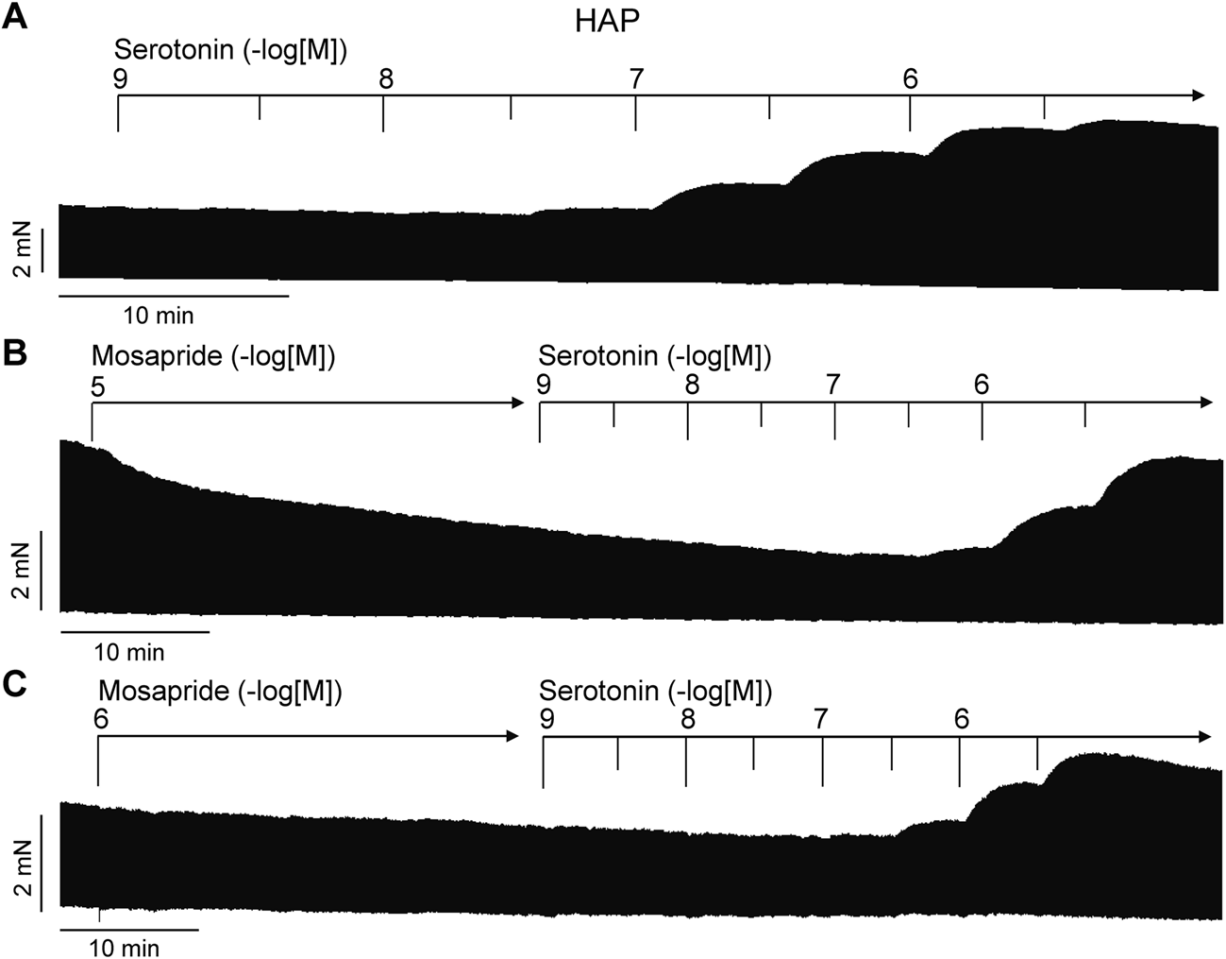
**A** Original recording of the concentration- and time-dependent positive inotropic effect of serotonin alone in millinewton (mN) in electrically stimulated human right atrial muscle strips. **B** Original recording of the concentration- and time-dependent positive inotropic effect of serotonin after application of 10 µM mosapride in millinewton (mN) in electrically stimulated human right atrial muscle strips. **C** Original recording of the concentration- and time-dependent positive inotropic effect of serotonin after application of 1 µM mosapride in millinewton (mN) in electrically stimulated human right atrial muscle strips. Horizontal bar indicates time axis in minutes (min). Concentrations of mosapride are given in negative logarithmic units in the horizontal arrow in the top

**Fig. 7:**
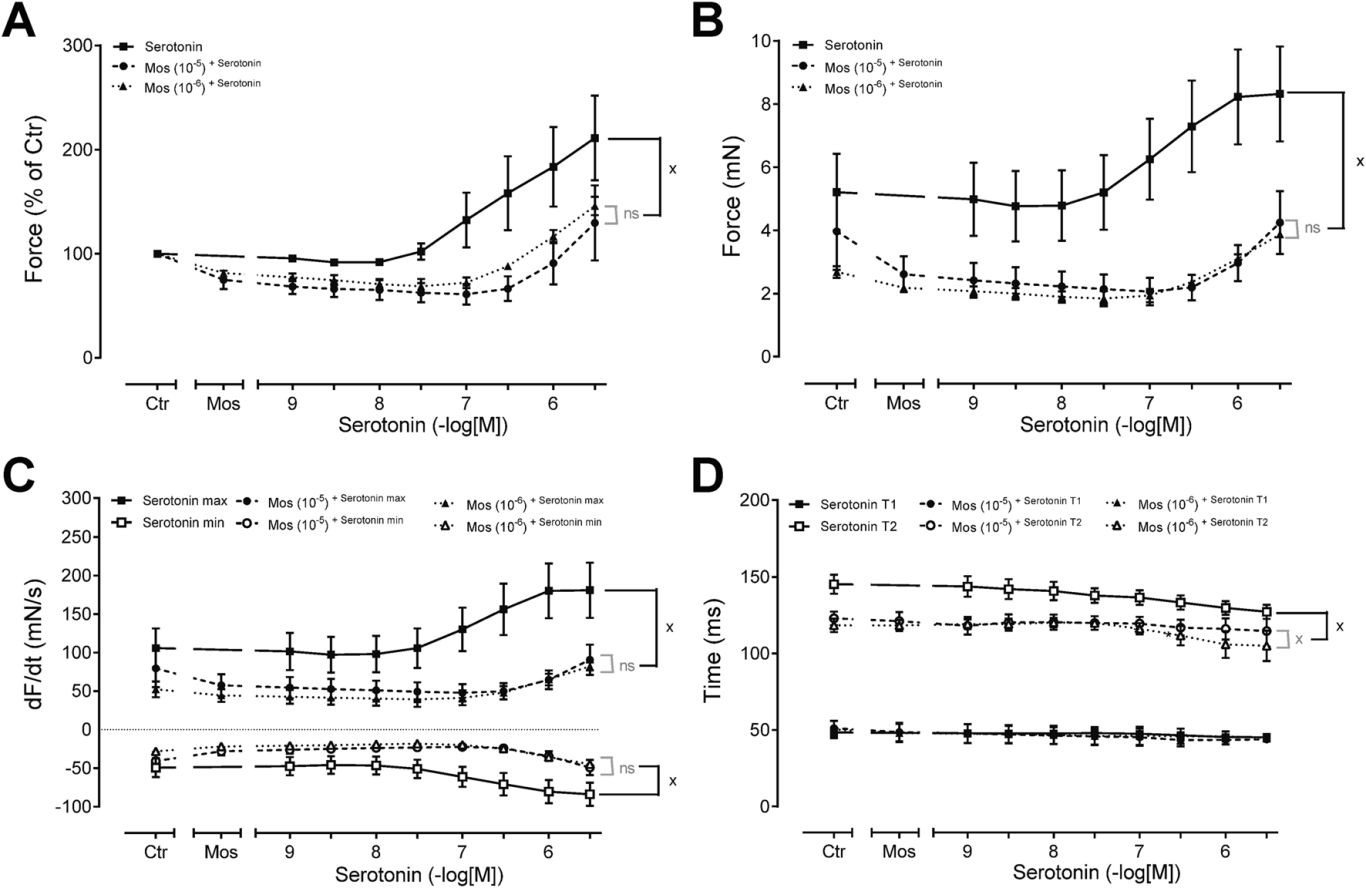
**A** force of contraction in % of pre-drug value. **B** Force of contraction in mN, **C** rate of contraction (dF/dt_max_), **D** rate of relaxation (dF/dt_min_). **D** Time to peak tension (T1), **D** time to relaxation (T2). Ordinates in panels **A** and **B** force of contraction in % of pre-drug value or millinewton (mN). Ordinates give rate of contraction and rate of relaxation in panel **C** in mN/s. Ordinates in panel **D** indicate milliseconds (ms). Significant difference versus serotonin alone is indicated by *x*. Numbers in brackets mean number of experiments. Abscissae in panels **A**–**D** indicate concentrations of serotonin in the presence of increasing concentrations of mosapride, both given in negative logarithms of molar concentrations. Significant difference versus control (Ctr; pre-drug value) or mosapride are indicated in * or #. Number of experiments were 3 to 9

Next, we wanted to test the effects of mosapride in the human heart similarly to those in mice, as shown in Fig. 4. To that end, we constructed concentration–response relationships for cumulatively applied serotonin alone or in the presence of increasing concentrations of mosapride. As seen in the original recording, while serotonin concentration-dependently increased (Fig. 6A), mosapride (1 µM: Fig. 6C; or 10 µM: Fig. 6B) alone did not increase but reduced the force of contraction. However, the concentration-dependent mosapride shifted the concentration-dependent effect of serotonin to the right (Fig. 6C, B). Several such experiments are summarised in Fig. 7. Mosapride shifted the effect of serotonin on the force of contraction in a concentration-dependent manner, as shown in mN (Fig. 7A) or % of the pre-drug value (Fig. 7B). Similarly, while serotonin alone raised the rate of tension development or rate of relaxation, these effects were concentration dependently reduced by mosapride (Fig. 7C). A similar pattern was seen at the time of relaxation (T2, Fig. 7D).

**Fig. 8:**
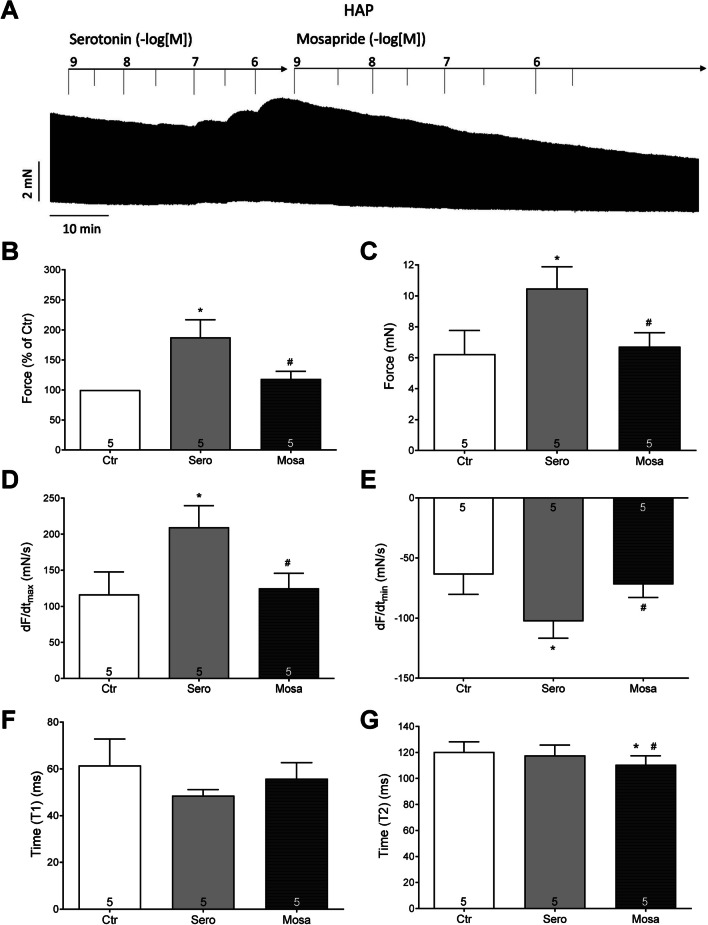
Effect of mosapride (1 µM) after serotonin (100 nM) on force of contraction in HAP. **A** An original recording of the concentration- and time-dependent positive inotropic effect of mosapride in millinewton (mN, vertical axis) in electrically stimulated HAP. First serotonin was applied then mosapride was given as a single concentration. Horizontal bar indicates time axis in minutes (min). **B** Force of contraction in % of pre-drug value. **C** Force of contraction in mN, **D** positive maximum rate of contraction (dF/dt_max_) and negative minimum rate of relaxation (**E**: dF/dt_min_). **F** Time to peak tension (T1) and time of relaxation (**G**: T2). Abscissae indicate molar concentrations of mosapride or serotonin in negative logarithms. Significant difference versus control (Ctr; pre-drug value) or serotonin are indicated in * or #. Numbers in brackets mean number of experiments

Next, we reversed the application of serotonin and mosapride. First, we established a concentration–response curve for serotonin, saturating cardiac 5-HT_4_ receptors (Fig. 8A). After that, increasing concentrations of mosapride were added (Fig. 8A). Here, we noted a negative inotropic effect of mosapride, displacing serotonin from receptors due to high concentrations of mosapride. This was seen in an original experiment (Fig. 8A). Data from several experiments are depicted in Fig. 8. Similar inotropic effects of mosapride were noted in absolute values for the force of contraction expressed in mN or % of the pre-drug value (Fig. 8B, C), for the rate of tension development and for the rate of relaxation (Fig. 8D). Moreover, we were interested in the effect of mosapride on the times of contraction. It turned out that mosapride reversed the effect of serotonin on the time of relaxation (T2, Fig. 8E).

**Fig. 9:**
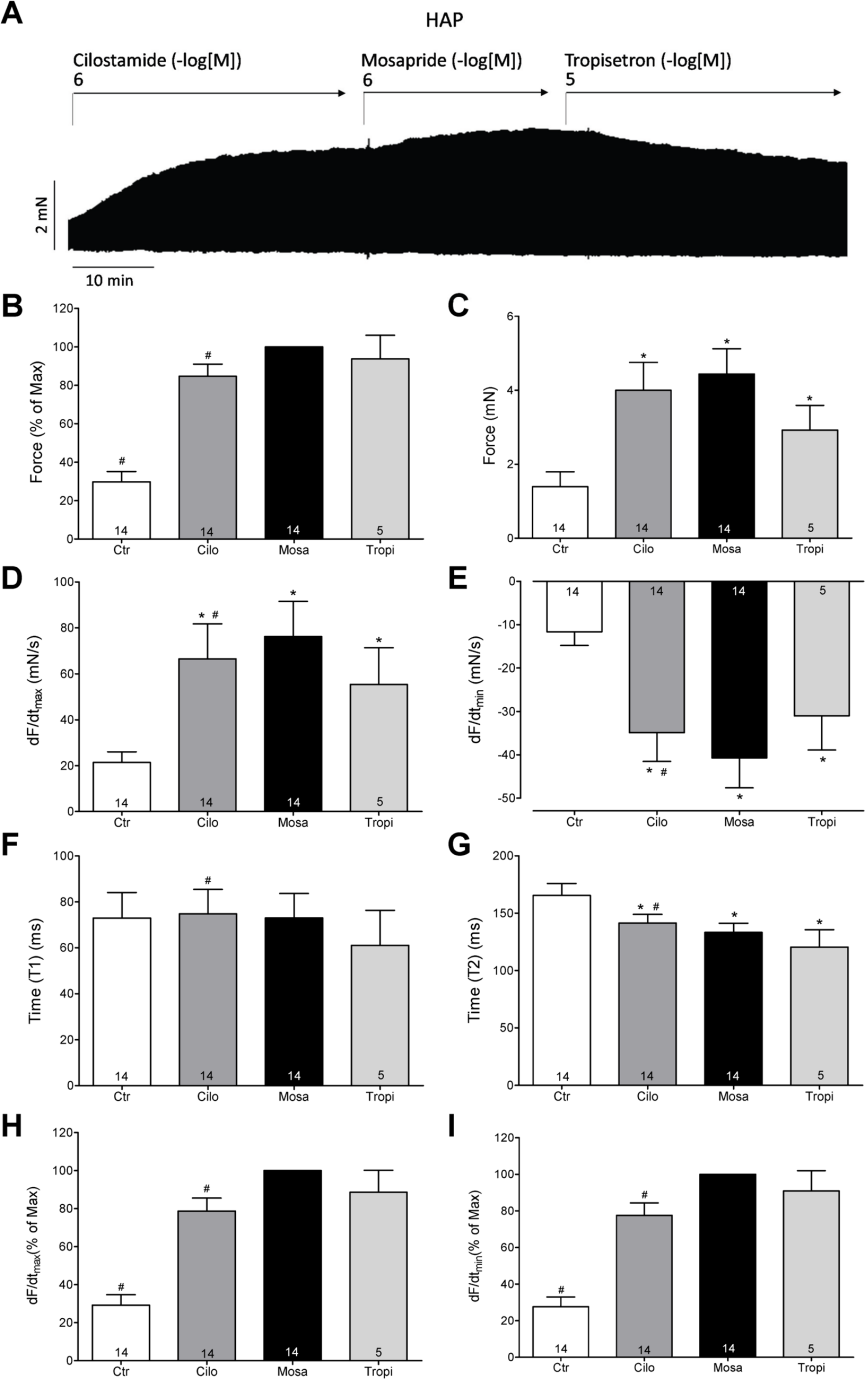
Effect of mosapride after cilostamide on force of contraction in HAP. **A** An original recording of the concentration- and time-dependent positive inotropic effect of mosapride in millinewton (mN, vertical axis) in electrically stimulated HAP. First cilostamide was applied then mosapride was given and finally tropisetron. Horizontal bar indicates time axis in minutes (min). **B** Force of contraction in % of pre-drug value. **C** Force of contraction in mN, **D** rate of contraction (dF/dt_max_), **E** rate of relaxation (dF/dt_min_), **F** time to peak tension (T1), **G** time to relaxation (T2). Ordinates in panels **A**, **C** and **B** force of contraction in millinewton (mN) or % of pre-drug values. Rate of contraction and rate of relaxation in panels **C** and **E** in mN/s. Ordinates in panel **E** time to peak tension (T1) and **F** gives time of relaxation (T2) in milliseconds (ms). **H** Rate of contraction (dF/dt_max_), **I** rate of relaxation (dF/dt_min_). For normalisation, we have defined the maximum effect of mosapride as 100%. Hence, the ordinates in panels **H** and **I** give force related to this maximum effect of mosapride. Significant difference versus control (Ctr; pre-drug value) or mosapride is indicated with * or #. Numbers in brackets mean number of experiments. In the bar diagram, pre-drug values (Ctr), the effect of cilostamide alone (Cilo), the effect of additional mosapride (Mosa) or additional tropisetron (Tropi) are indicated. The *p*-values of panel **B** of Mosa vs. Ctr to *p* ≤ 0.0001 or Mosa vs. Cilo to *p* = 0.0319 or Mosa vs. Tropi to* p* = 0.3805. The *p*-values of panel **C** of Ctr vs. Cilo and Ctr vs. Mosa to *p* ≤ 0.0001 or Ctr vs. Tropi to *p* = 0.0016 and Mosa vs. Cilo to *p* = 0.057 or Mosa vs. Tropi to *p* = 0.8059. The *p*-values in panel **D** amount for Ctr vs. Cilo to *p* = 0.0006 or Ctr vs. Mosa *p* ≤ 0.0001 or Ctr vs. Tropi *p* = 0.0177 and Mosa vs. Cilo *p* = 0.0303 or Mosa vs. Tropi *p* = 0.9769. The *p*-values in panel **E** amount for Ctr vs. Cilo to *p* = 0.0001 or Ctr vs. Mosa *p* ≤ 0.0001 or Ctr vs. Tropi *p* = 0.0024 and Mosa vs. Cilo *p* = 0.0096 or Mosa vs. Tropi *p* = 0.9924. The *p*-values in panel **F** amount for Ctr vs. Cilo to *p* = 0.836 or Ctr vs. Mosa and Ctr vs. Tropi *p* ≥ 0.999 and Mosa vs. Cilo *p* ≤ 0.0001 or Mosa vs. Tropi *p* = 0.8937. The *p*-values in panel **G** amount for Ctr vs. Cilo to *p* = 0.027 or Ctr vs. Mosa *p* = 0.0023 or Ctr vs. Tropi *p* = 0.0099 and Mosa vs. Cilo *p* = 0.0005 or Mosa vs. Tropi *p* = 0.2754. The *p*-values in panel **H** amount for Mosa vs. Ctr to *p* ≤ 0.0001 or Mosa vs. Cilo to *p* = 0.0024 or Mosa vs. Tropi to *p* ≥ 0.999. The *p-*values in panel **I** amount for Mosa vs. Ctr to *p* ≤ 0.0001 or Mosa vs. Cilo to *p* = 0.0014 or Mosa vs. Tropi to *p* ≥ 0.999

While mosapride alone failed to raise the force of contraction but reduced the force of contraction (Fig. 6), when force had been raised by cilostamide, a phosphodiesterase III inhibitor (which we have used before to raise force in HAP: e.g. Gergs et al. 2024), then additionally applied mosapride exerted a positive inotropic effect that could be reduced by receptor antagonists. The original recording is depicted in Fig. 9A. Data from several experiments are depicted in Fig. 9. Cilostamide increased force, and this effect was amplified by mosapride and antagonised by tropisetron, here used as an inhibitor of human 5-HT_4_ serotonin receptors, in HAP used before by others (Kaumann et al. 1990) (Fig. 9C). Similar inotropic effects of mosapride were noted in absolute values for the rate of tension development and the rate of relaxation (Fig. 9D).

Moreover, we were interested in the effect of mosapride on the times of contraction. Cilostamide and additional mosapride did not alter the time to peak tension (Fig. 9F). Cilostamide itself reduced the time of relaxation (T2, Fig. 9G); this effect was accentuated by mosapride, but was not reversed by additional tropisetron (Fig. 9G). Finally, we calculated the percent increase in the rate of tension development and the rate of relaxation. In other words, pre-drug values before cilostamide (Fig. 8A) were arbitrarily set to 100%. If this normalisation procedure is used, it becomes more apparent that cilostamide alone increased the rate of tension development and this was accentuated by mosapride (Fig. 9H). Likewise, cilostamide alone increased the rate of tension relaxation and this was accentuated by mosapride (Fig. 9I).

**Fig. 10:**
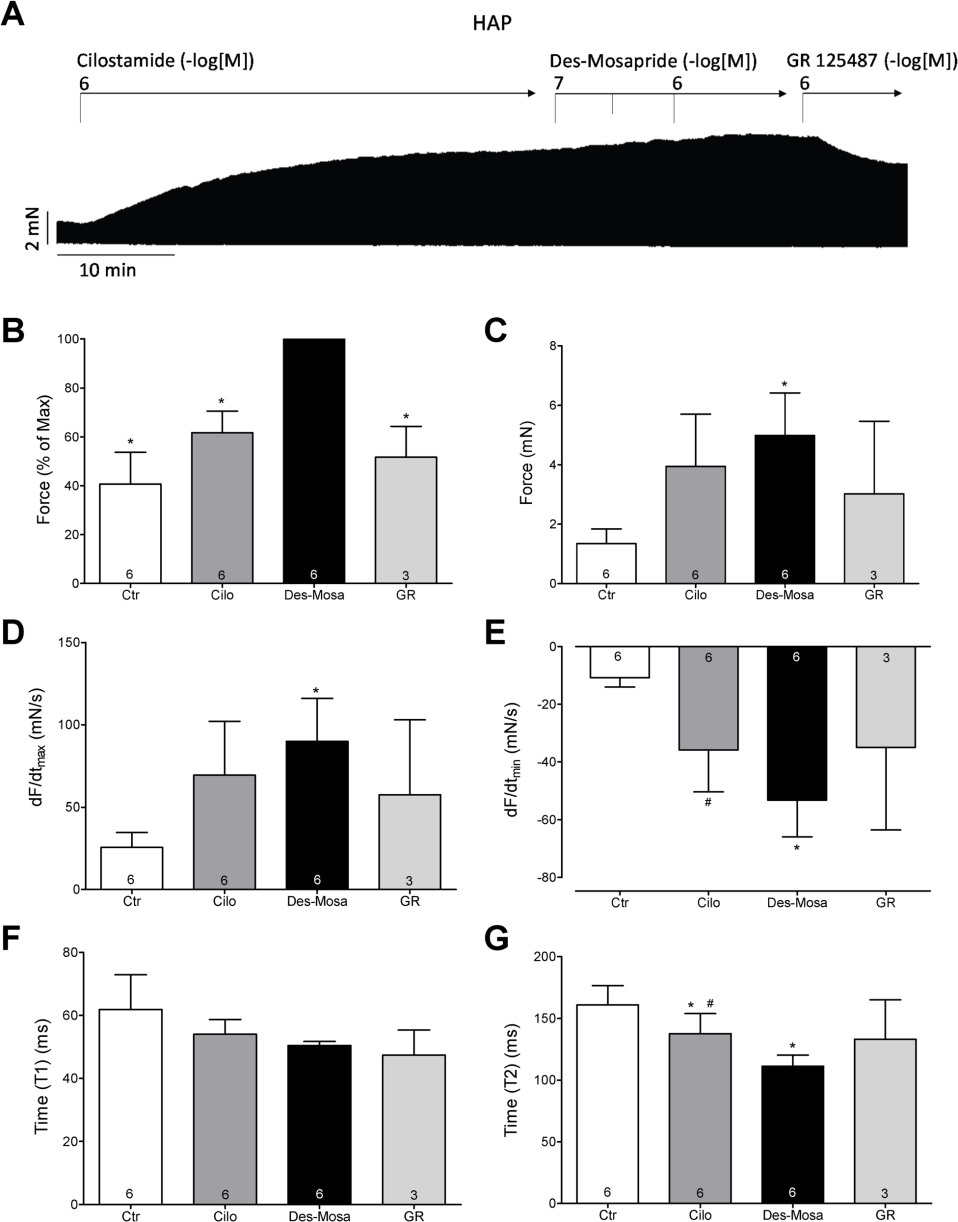
Original recording of the concentration- and time-dependent positive inotropic effect of des-4-fluoro-benzyl-mosapride (Des-Mosa) in millinewton (mN) in electrically stimulated HAP. First cilostamide (Cilo) was applied then des-4-fluoro-benzyl-mosapride was cumulatively applied thereafter GR125487 (GR). Horizontal bar indicates time axis in minutes (min). Ordinates in panels **A**, **B** and **C** force of contraction in milliNewton (mN) or % of maximum effect of value to Des-Mosa. Rate of contraction and rate of relaxation in panels **D** and **E** in mN/s. Ordinates in panel **F** give time to peak tension (T1) and time of relaxation (T2) in panel **G** in milliseconds (ms). Abscissae indicate molar concentrations of des-4-fluoro-benzyl-mosapride in negative decadic logarithms. Significant difference versus Des-Mosa or Ctr (pre-dug value) is indicated by # or *. Numbers in brackets mean number of experiments

Finally, the question arose of how the primary metabolite of mosapride would affect the force of contraction in HAP. We observed the same pattern as with the mother compound, mosapride. Specifically, as seen in the original recording, cilostamide slowly increased the force of contraction (Fig. 10A). After that, mosapride concentration dependently increased the force of contraction; this increase could be antagonised by GR125487, an antagonist at human cardiac 5-HT_4_ serotonin receptors (e.g. Gergs et al. 2010, 2013) (Fig. 10A). Several similar experiments were then summarised. We report similar positive inotropic effects of des-mosapride for the force of contraction expressed in mN or % of the maximum value (Fig. 10B, C) for the rate of tension development and the rate of relaxation (Fig. 10D) in absolute values by mosapride (Fig. 10E). Moreover, we were interested in the effect of mosapride on the times of contraction. It turned out that mosapride reversed the effect of serotonin not on time to peak tension (T1, Fig. 10F) but on time of relaxation (T2, Fig. 10G).

## Discussion

### Main new findings

The primary new finding is that mosapride can function as a partial functional agonist at 5-HT_4_ receptors in transgenic mouse hearts and HAP.

### Mechanism of mosapride

We suggest that mosapride increased force and beating rate as an agonist at cardiac human 5-HT_4_ receptors because mosapride only increased the force of contraction in the left atrium from 5-HT_4_-TG and not in WT. Because the maximum inotropic effect of mosapride in atrial preparations of 5-HT_4_-TG could be further stimulated by additional serotonin, we tentatively conclude that mosapride acts here as a partial agonist. Likewise in HAP, mosapride could act as an agonist and could increase force of contraction via 5-HT_4_ receptors. However, this positive inotropic effect of mosapride was only seen in the presence of cilostamide, a phosphodiesterase III inhibitory drug. In contrast, in the absence of cilostamide (Fig. 6), mosapride reduced force of contraction. This could mean that mosapride can also reduce force of contraction via 5-HT_4_ receptors. Conceivably, mosapride might in this case act as an inverse agonist at 5-HT_4_ receptors.

### Role of phosphorylation of regulatory proteins

The general assumption is that 5-HT_4_ receptor stimulation increases the phosphorylation of protein substrates for cAMP-dependent protein kinase (Fig. 1B). We and others described that serotonin via 5-HT_4_ receptors can increase the phosphorylation state of phospholamban (Gergs et al. 2009, Neumann et al. 2019, 2023b). These phosphorylations can partly explain why mosapride increased the force in atrial preparations from 5-HT_4_-TG.

Mosapride functioned as an agonist at 5-HT_4_ receptors in the isolated HAP. We conclude this because the positive inotropic effect of mosapride (in the presence of cilostamide) is antagonised by 5-HT_4_ antagonists like tropisetron and GR 125487. Moreover, mosapride, as in 5-HT_4_-TG, acts as a partial agonist. Mosapride shifted the concentration–response curve (on the force of contraction) of serotonin to the right in HAP, which was expected from a 5-HT_4_ antagonist. Moreover, mosapride attenuated the positive inotropic effect of serotonin. Such partial agonisms in the human heart are not without precedence for 5-HT_4_ receptor agonists. For inotropy, cisapride and metoclopramide are partial agonists in 5-HT_4_-TG but also in HAP (Chai et al. 2012, Keller et al. 2018, Neumann et al. 2021b). Of note, cisapride and metoclopramide are structurally similar to mosapride, further supporting our conclusion.

### Species differences

A significant merit of this study is that we used a small animal model (5-HT_4_-TG) to test for the inotropic effects of mosapride. In previous papers, rats, rabbits or dogs were used to study the cardiac effects of mosapride. While these studies are well suited to investigate the effects of mosapride via potassium channels (notably hERG), they are not useful to study the effects of mosapride or its metabolites on 5-HT_4_ receptors; dog, rabbit and rat hearts (and WT mouse hearts) do not contain functional 5-HT_4_ receptors that couple to force of contraction (reviewed in Neumann et al. 2017, 2023a). One could study mosapride in porcine hearts, but pigs are more expensive than mice; the sequence of the 5-HT_4_ receptor is similar, but not identical, in pigs and humans. In 5-HT_4_-TG, we encountered the same sequence as in human hearts because we chose to overexpress the human 5-HT_4_ receptor.

Moreover, our comparative study on 5-HT_4_-TG and HAP showed another intriguing species difference. Whereas mosapride alone was an agonist in the atrial preparations of 5-HT_4_-TG, mosapride alone was ineffective in raising the force of contraction in HAP. At least two reasons could explain these differences between mice and humans, which are not mutually exclusive. First, the overexpression of 5-HT_4_ receptor is so high in 5-HT_4_-TG that even an inverse agonist can stimulate the receptor. Alternatively, the signal transduction proteins (e.g. Gs, AC, PKA, Fig. 1A) are so different between mice and humans that stimulation of mosapride leads to different steps in signal transduction. It would be interesting to address this issue in subsequent work. There is also evidence in the gastroenterological tract that mosapride can act as a partial agonist (Yoshida and Ito 1994).

Notably, mosapride acted more potently to raise force in transgenic mice than in the human atrium. This is consistent with our previous work on cisapride, prucalopride or metoclopramide (Keller et al. 2018, Neumann et al. 2021b). We assume this is due to the much higher level of expression of 5-HT_4_ receptors in mouse hearts than in human hearts (Neumann et al. 2021a). We argue that the 5-HT_4_-TG offer the possibility of amplifying any effect of agonists at 5-HT_4_ receptors. On the other hand, if a putative 5-HT_4_-agonist does not act in 5-HT_4_-TG, it is unlikely to work as an agonist in human tissue. In isolated rabbit hearts, 10 μM of mosapride prolonged the QT interval (Kii and Ito 2002). This was explained by the inhibition of potassium currents.

In this study (Fig. 3D), we did not detect a reduction in time of relaxation, which is typical of 5-HT4-mediated effects by serotonin alone (Gergs et al. 2009, 2010). We speculate that any shortening via 5-HT4 receptor stimulation is offset by the inhibition of potassium channels that prolong the duration of the contraction. In HAP, we did not observe a further reduction in the time of relaxation (Fig. 9G) compared to cilostamide. We assume that cilostamide had already maximally reduced the time of relaxation, and thus no additional shortening by mosapride was detectable. Alternatively, one might speculate that in the human atrium, some degree of inhibition of the potassium channels might have led to the mixed effect of mosapride.

On the other hand, as seen in Fig. 10G, Des-Mosa reduced the time of relaxation, even in the additional presence of cilostamide. A potential explanation for the different effects of mosapride and De-Mosa on the time of relaxation might reside in the following: De-Mosa might not be able to inhibit potassium channels in human hearts and might solely act on 5-HT4 receptors, therefore reducing the time parameters. However, one would need to know how Des-Mosa acts on the action potential in HAP to confirm this assumption.

### Effects on the beating rate

We assume that, like 5-HT, mosapride stimulated 5-HT_4_ receptors in the right atrium of 5-HT_4_-TG. This conclusion is based on the observation that the effect is absent in the right atrium from the WT. Mosapride acted like various other agonists (cisapride, prucalopride, metoclopramide) as a partial agonist compared to the chronotropic effect of 5-HT (Keller et al. 2018, Neumann et al. 2021b). The data on the beating rate might have clinical relevance because it is rarely possible to obtain sinus node cells from patients and to test mosapride in such spontaneously beating cells. However, our data predict that mosapride can lead to tachycardia in human hearts. In contrast, we cannot rule out from our contraction data in the HAP that mosapride might act as an antagonist in the human sinus node. This should result in bradycardia, because serotonin is present in the human atrium in thrombocytes and continuously forms in the human atrium.

### Clinical relevance

To our knowledge, this is the first study on the effects of mosapride on the force of contraction in isolated HAP. Hence, this is the first report of any effect of mosapride in the human heart and its mediation via the 5-HT_4_ receptor, which adds to the clinical knowledge about mosapride.

We predict that tachycardia after treatment with mosapride in patients could be blocked by tropisetron, an approved drug. However, this prediction must be confirmed in a clinical study. On the other hand, if mosapride mainly acts as an antagonist, mosapride should reduce the beating rate. In a study of healthy volunteers, 10 mg mosapride per os changed the spectral form of the ECG. The QT was shorter after the mosapride, but not significantly different. The heart rate was lower with mosapride, but this was also not significant (Endo et al. 2002). Peak therapeutic plasma levels of mosapride when taking 40 mg per mouth in healthy volunteers amounted to 282 ng/ml (0.67 µM). Hence, the concentrations tested here in vitro for mosapride might be achieved in humans. Moreover, in intoxications, much higher plasma levels of mosapride are expected. Mosapride is degraded mainly by CYP3A4 (Katoh et al. 2003). This enzyme is inhibited by antifungal azoles and some antibiotics, such as erythromycin.

A Japanese study found that the metabolism of mosapride in healthy volunteers was impaired by erythromycin (Katoh et al. 2003). This drug–drug interaction increased plasma concentrations of mosapride from about 42 to about 67 ng/ml and prolonged the half-life of mosapride (Katoh et al. 2003). In contrast to cisapride, which blocks ventricular potassium ion channels in humans, mosapride is 400–1000-fold less potent than cisapride in inhibiting these channels (Katoh et al. 2003).

Mosapride and its metabolites are primarily eliminated by the kidneys (Katoh et al. 2003). As kidney function declines with ageing, the elimination half-life of mosapride is probably augmented in the elderly. This kinetic behaviour is problematic, as the cumulation of mosapride and an increase in plasma mosapride concentration are expected. Seniors are also more likely to develop atrial fibrillation in the first place. Moreover, many drugs would inhibit the metabolism of mosapride, further increasing plasma concentrations and potential cardiac side effects of mosapride. One could question the relevance of our findings regarding cilostamide and mosapride. Usually, patients take mosapride in the absence of a phosphodiesterase inhibitor. However, in heart failure patients, the phosphodiesterase inhibitors pimobendan, milrinone or levosimendan are sometimes given. Moreover, many patients drink coffee, which contains the phosphodiesterase inhibitor caffeine.

Finally, our work adds to our knowledge by showing that the primary metabolite of mosapride is active at 5-HT_4_ receptors. From this, one might predict that the cardiac action of mosapride may last longer than predicted from the half-life of mosapride because, after that, the metabolite might still be active in humans.

### Limitations of the study

One can argue that we have not tested the effects on the sinus node of man directly. Such a study would require access to a human pacemaker. Such studies were beyond the scope of this initial study. Furthermore, due to a lack of access to that tissue, we did not have the opportunity to study contractility in human ventricle tissue.

Figure 6 shows that mosapride alone could decrease the force of contraction in the HAP. The reasons for this negative inotropic effect have not been studied here. An attractive hypothesis is that mosapride might act as an inverse agonist at the 5-HT_4_ serotonin receptors in HAP. We have shown that in human HAP, serotonin can be produced (Gergs et al. 2017). This serotonin might continuously stimulate HAP, which may be antagonised by mosapride.

In summary, we can now address the hypotheses raised in the ‘Introduction.’ First, mosapride raised the force of contraction and beating rate in atrial preparations from 5-HT_4_-TG but not from WT. Second, mosapride and its primary metabolite elevated the force of contraction in the HAP via 5-HT4 receptors.

## Data Availability

No datasets were generated or analysed during the current study.
